# Interface Bond Improvement of Sisal Fibre Reinforced Polylactide Composites with Added Epoxy Oligomer

**DOI:** 10.3390/ma11030398

**Published:** 2018-03-07

**Authors:** Mingyang Hao, Hongwu Wu, Feng Qiu, Xiwen Wang

**Affiliations:** 1 The Key Laboratory of Polymer Processing Engineering of Ministry of Education, South China University of Technology, Guangzhou 510640, China; hmyang1992@163.com (M.H.); qiufeng868@gmail.com (F.Q.); wangxwen0623@163.com (X.W.); 2 National Engineering Research Center of Novel Equipment for Polymer Processing, South China University of Technology, Guangzhou 510640, China

**Keywords:** polymer-matrix composites, natural fiber reinforcement, interface/interphase, microstructural analysis, crystallization behavior, rheological behavior

## Abstract

To improve the interfacial bonding of sisal fiber-reinforced polylactide biocomposites, polylactide (PLA) and sisal fibers (SF) were melt-blended to fabricate bio-based composites via in situ reactive interfacial compatibilization with addition of a commercial grade epoxy-functionalized oligomer Joncryl ADR^@^-4368 (ADR). The FTIR (Fourier Transform infrared spectroscopy) analysis and SEM (scanning electron microscope) characterization demonstrated that the PLA molecular chain was bonded to the fiber surface and the epoxy-functionalized oligomer played a hinge-like role between the sisal fibers and the PLA matrix, which resulted in improved interfacial adhesion between the fibers and the PLA matrix. The interfacial reaction and microstructures of composites were further investigated by thermal and rheological analyses, which indicated that the mobility of the PLA molecular chain in composites was restricted because of the introduction of the ADR oligomer, which in turn reflected the improved interfacial interaction between SF and the PLA matrix. These results were further justified with the calculation of activation energies of glass transition relaxation (∆*E*_a_) by dynamic mechanical analysis. The mechanical properties of PLA/SF composites were simultaneously reinforced and toughened with the addition of ADR oligomer. The interfacial interaction and structure–properties relationship of the composites are the key points of this study.

## 1. Introduction

Due to increasing concerns about environment and sustainability issues, natural fibers have attracted extensive attention for their renewable and sustainable nature. The development of natural fiber-reinforced polymer composites has attracted wide attention both in academia and industry, due to their low cost, light weight, ability to be recycled, good structural properties, and so on [1,2,3,4].

The mechanical properties of fiber-reinforced polymer composites are influenced not only by the intrinsic properties of fibers and polymer matrix, but also by the interfacial properties of the composites [5]. The stress is transferred from the polymer matrix to the fibers via the interface of the composite under load-bearing. Therefore, a fundamental study of the fiber–matrix interface is critical to the development of fiber-reinforced polymer composites. Numerous studies have proved that a good interfacial adhesion is of great importance for obtaining good mechanical properties of composites materials [6]. Good interfacial compatibility between the fibers and the polymer matrix will result in efficient stress transferring from matrix to the fiber, and thus improve the mechanical performance of composites. For natural fiber-reinforced polymer composites, the interfacial compatibility between natural fibers and matrix is generally poor due to the hydrophilic nature of natural fiber and the hydrophobic nature of polymer matrix. In natural fiber-reinforced polymer composites, the two phases are mechanical and/or chemically combined, and the fiber–matrix interface can be considered as a diffusion or reaction zone. Poor chemical and physical interfacial interaction between natural fiber and polymer matrix are regarded as the most important mechanisms of bond failure.

To improve the interfacial interaction of natural fiber-reinforced polymer composites, many surface modification methods were applied to natural fibers to improve the compatibility between fibers and matrix [7]. It had been proved that chemical treatments of natural fiber surface were an effective way to improve the interfacial compatibility and adhesion of natural fiber-reinforced polymer composites [8]. Several types of chemical treatment methods were developed for surface modification of natural fiber, including silane treatment [9,10], acetylation treatment [11], benzoylation treatment [12], acrylation and acrylonitrile grafting [13,14], dopamine treatment [15], N-methylol acrylamide grafting [4], and so on. Alkali treatment with sodium hydroxide is one of the most widely used chemical treatment methods for natural fibers [16,17]. It can remove the lignin, natural fats, waxes, and impurities from natural fiber surfaces. Therefore, the surface roughness of natural fibers is improved and more reactive functional groups and other reactive functional groups are revealed on the fiber surface. Alkali treatment with sodium hydroxide has been widely used before other chemical treatment is performed in natural fibers surface modification to promote the interfacial interaction [18]. These chemical treatment methods could improve the interfacial adhesion of natural fibers and polymer matrices, but sometimes weaken the fiber strength itself at the same time. Furthermore, these methods generally require a long time for soaking the natural fibers in the corresponding solution. They are inefficient. Meanwhile, the organic solvents used in these methods are usually not friendly to the environment. Reaction processing can improve the interfacial compatibility of composites via in-situ reaction during melt-blending processing, thus it is a promising method for preparing natural fiber-reinforced polymer composites without the disadvantages of chemical treatment methods.

Joncryl ADR^®^-4368 is a kind of commercial grade multi-epoxy-functionalized styrene-acrylic oligomer (ADR) [19,20,21]. We consider that ADR has the potential to be an efficient reactive compatibilizer in natural fiber reinforced polymer composites. In this study, polylactide and sisal fibers were used to fabricate bio-based natural fiber reinforced polymer composites. To improve the interfacial adhesion of PLA/sisal fibers composites, polylactide resin and sisal fibers were melt-blended via in situ reactive interfacial compatibilization with the addition of ADR oligomer. The in-situ reaction processing method has the possibility of bonding PLA molecule chains onto the fiber surface via interfacial reaction of the functional groups on the natural fibers’ surface and the end group of PLA. The phase morphology, thermal behaviors, rheological behaviors, dynamic mechanical analysis, and tensile properties of PLA/sisal fiber composites were investigated. The interfacial interaction and structure–properties relationship of composites are key points of this study.

## 2. Experimental Section

### 2.1. Materials

The PLA (trade name 4032D), a semi-crystalline extrusion grade with 1.2–1.6% D-isomer lactide and density of 1.25 g/cm^3^, from Nature Works LLC (Minnetonka, MN, USA) was used. It was dried in an air-dry oven at 80 °C for 8 h before use. The chopped sisal fibers (6 mm) were purchased from Dongfang Sisal Co. (Guangdong, China) and the properties of the fiber provided by the supplier are shown in Table 1. Joncryl ADR^®^-4368 was provided by Shanghai Kingpont Chemical Company, China P. R.

### 2.2. Preparation of the Composites

Sisal fibers were soaked in sodium hydroxide solution (5 wt %) for 1 h to remove lignin, pectin, and waxy substances on sisal fibers surface, then were vacuum dried at 80 °C for 8 h. Melt blending of PLA, sisal fibers (SF), and ADR was performed using an internal mixer (RTOI-55, Potop, Guangzhou, China) at 200 °C for 5 min with a roller speed of 80 rpm. A series of PLA/SF and PLA/SF/ADR composites with different ADR addition and different SF content were prepared. Then, the obtained mixtures were compression-molded at 200 °C for 3 min under 10 MPa into standard specimens for rheological dynamic frequency sweep tests and mechanical tests. At the same time, to characterize the internal reaction of the composites melt, rheological dynamic time sweep tests were performed. The PLA/SF/ADR composites samples were prepared using an internal-mixer at a relatively low temperature (180 °C) for 2 min without any further melt processing. The dynamic time sweep tests were executed at a melt mixing temperature of 200 °C.

### 2.3. Measurements of Mechanical Properties

Notched Izod impact tests were performed following the ISO 180, using a 5.5 J pendulum at room temperature. The tensile tests were carried out on a universal tensile testing machine (Instron 5566, Norwood, MA, USA) according to ISO 527-2 with a crosshead speed of 2 mm/min. At least five specimens for each composite were tested. The average values were calculated and the standard errors were obtained as well.

### 2.4. Morphological Characterization

The specimens after the tensile tests and Izod impact tests were used for morphological characterization. The tensile and impact fracture surfaces morphology of the composites were recorded by scanning electron microscopy (SEM, FEI Quatan 250, Thermo Fisher Scientific, Hillsboro, OR, USA). Fracture surfaces were sputtered with gold before SEM observation to provide enhanced conductivity.

### 2.5. FTIR Measurement

In order to estimate the interfacial bonding between PLA and sisal fibers via reaction with ADR oligomer during the melt-blending processing, the sisal fibers were obtained from composites by Soxhlet extraction using dichloromethane as solvent and dried at 80 °C for 8 h in a vacuum oven, and then ground into powder for FTIR analysis. The FTIR spectroscope (Nexus 670, Thermo Nicolet Co. Ltd., Waltham, MA, USA, KBr powder) was used to characterize the extracted fibers over a range of 4000–400 cm^−1^. For comparison, FTIR characterization of PLA resin was also carried out.

### 2.6. Thermal Analysis

The thermal behavior of the composites was studied using a differential scanning calorimeter (DSC-204C, NETZCH, Selb, Germany). For isothermal melt crystallization, the samples were heated from 30 °C to 190 °C and held for 5 min at 190 °C to eliminate the thermal history, and then cooled down to 110 °C at a rate of 50 °C/min and held for a period of time until the isothermal crystallization was complete. The isothermal crystallization behaviors at different crystallization temperature were also performed. For non-isothermal melt crystallization, the samples were first heated from room temperature to 190 °C and held for 5 min to eliminate the thermal history, and then cooled back to 30 °C at a rate of 10 °C/min. After 2 min in 30 °C, the second heating scan from 30 °C to 190 °C at 10 °C/min was performed. The cooling-crystallization of PLA/SF/ADR composites at 5 °C/min was also carried out. In the whole process, all samples were kept under nitrogen flow of 25 mL/min.

### 2.7. Rheological Characterization

Viscoelastic behavior of composites was measured by a dynamic oscillatory rheometer (Anton paar, MCR 302, Graz, Austria), equipped with a plate diameter of 25 mm and a gap of 1 mm parallel plate geometry. The following tests were performed: (a) dynamic small amplitude oscillatory frequency sweep tests from 0.0628 rad/s to 628 rad/s at 180 °C with a strain amplitude of 1%; and (b) dynamic time sweep test using 1% strain amplitude and an angular frequency of 10 rad/s at 200 °C.

### 2.8. Dynamic Mechanical Analysis (DMA) Testing

The DMA measurements (DMA-242 E, NETZSCH, Selb, Germany) were carried out at 0.1, 0.5, 1.0, 5, and 10 HZ, respectively, under a heating rate of 2 °C/min, using three point bending 40 mm measurement method. The temperature range was from 30 °C to 110 °C.

## 3. Results and Discussion

### 3.1. Morphology Analysis

**Figure 1:**
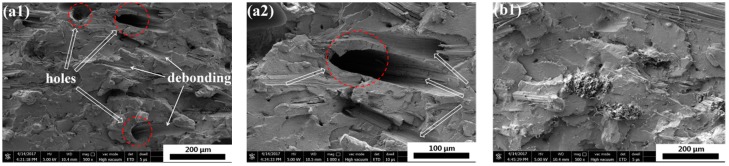

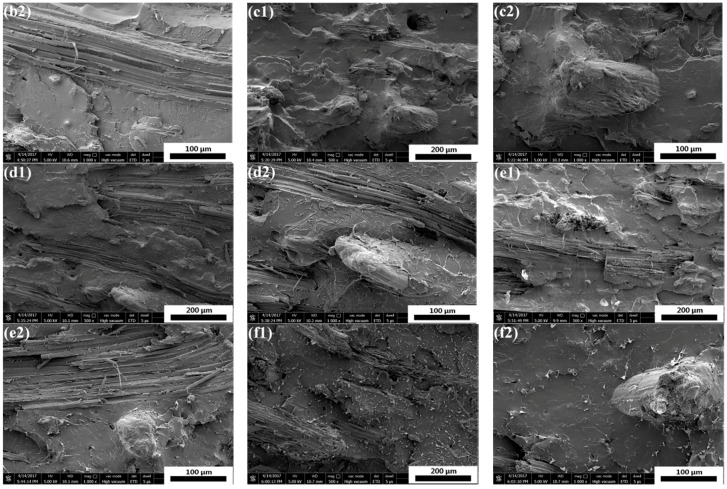
The impact fracture surfaces of polylactide (PLA)/sisal fibers (SF) (80/20) (**a1**,**a2**) and PLA/SF/ADR composites with different ADR content: (80/20/0.2) (**b1**,**b2**); (80/20/0.4) (**c1**,**c2**); (80/20/0.6) (**d1**,**d2**); (80/20/0.8) (**e1**,**e2**); (80/20/1.0) (**f1**,**f2**).

**Figure 2:**
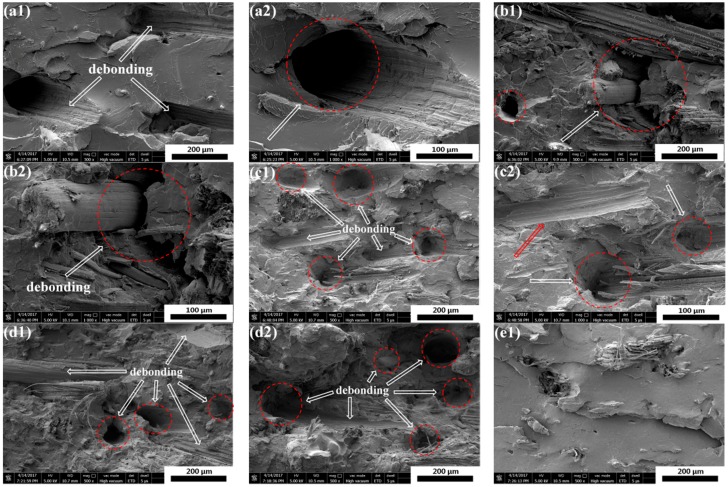

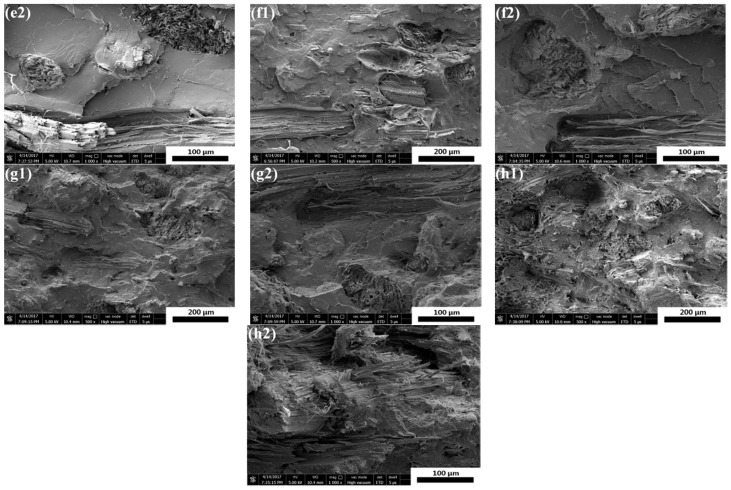
The impact fracture surfaces of PLA/SF composites (90/10) (**a1**,**a2**); (80/20) (**b1**,**b2**); (70/30) (**c1**,**c2**); (60/40) (**d1**,**d2**) and PLA/SF/ADR composites (90/10/0.6) (**e1**,**e2**); (80/20/0.6) (**f1**,**f2**); (70/30/0.6) (**g1**,**g2**); (60/40/0.6) (**h1**,**h2**) with different SF content.

**Figure 3:**
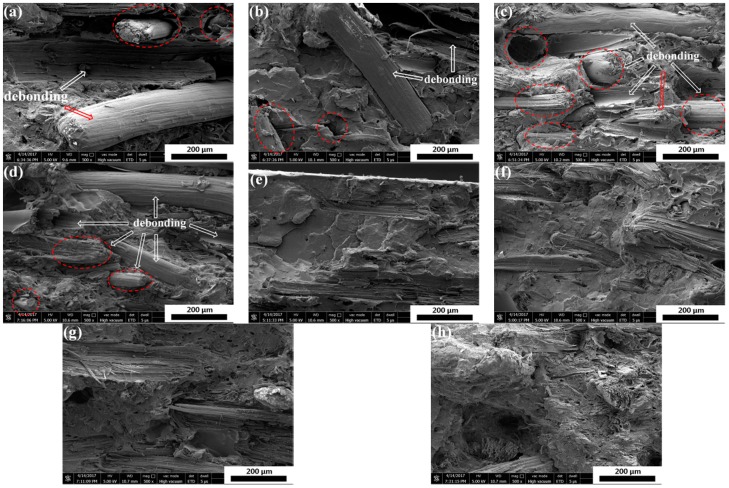
The tensile fracture surfaces of PLA/SF composites (90/10) (**a**); (80/20) (**b**); (70/30) (**c**); (60/40) (**d**) and PLA/SF/ADR composites (90/10/0.6) (**e**); (80/20/0.6) (**f**); (70/30/0.6) (**g**); (60/40/0.6) (**h**) with different SF content.

The impact fracture surfaces of PLA/SF and PLA/SF/ADR composites with constant 20 wt % SF content and different ADR addition are shown in Figure 1. It can be seen from Figure 1a, that for PLA/SF composites many fibers were directly pulled out from PLA matrix, and many holes were formed in the fracture surfaces, reflecting poor interfacial adhesion between the PLA matrix and sisal fibers. However, the addition of ADR into composites during melt-blending processing could effectively counteract this phenomenon, as shown in Figure 1b–f for which the ADR content varied from 0.2 wt % to 1.0 wt %. For PLA/SF/ADR composites, the fibers were tightly connected with matrix and it was observed that more fibers were broken up or torn off in composites with ADR addition compared with that of PLA/SF composites. These phenomena demonstrated the improved interfacial adhesion of PLA/SF/ADR composites via addition of ADR compared with that of PLA/SF composites. In order to further confirm this result, the impact fracture surfaces of PLA/SF composites and PLA/SF/ADR composites with different SF content are presented in Figure 2. For PLA/SF composites, similar phenomena were observed. Many fibers were directly pulled out from PLA matrix and many holes were formed in the fracture surfaces, reflecting poor interfacial adhesion (Figure 2a–d). It was also observed that the more SF was added, the more fracture defects occurred. The presence of ADR improved the interfacial adhesion between SF and PLA matrix (Figure 2e–h). Furthermore, the tensile fracture surfaces of PLA/SF composites and PLA/SF/ADR composites with different SF content are shown in Figure 3. It can be seen from Figure 3a–d that the sisal fibers were directly pulled out from the PLA matrix in PLA/SF composites, which impairs the reinforcing effect of SF during tensile tests. However, for PLA/SF/ADR composites, the improved interfacial adhesion between the SF and PLA matrix caused many fibers to be broken up or torn off in the tensile fracture surface, which indicated that the fibers bore the load in the tensile tests.

**Figure 4:**
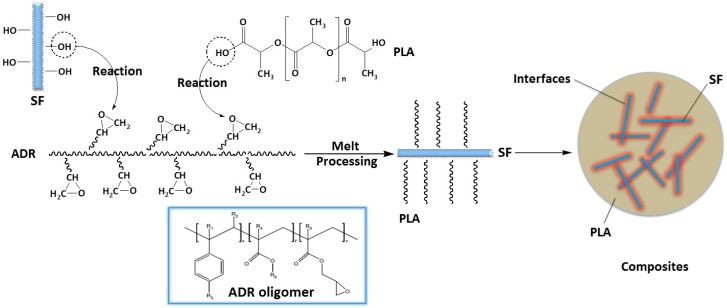
Illustration of interfacial compatibilization between PLA and SF via in situ reaction with ADR oligomer during the melt-blending processing.

Many modification methods of PLA resin are based on the reaction ability of the end group of PLA molecule chain, that are hydroxyl and carboxyl, by which the typical works are reaction toughening of PLA [22,23,24,25,26]. The presence of hydroxyl groups on the natural fiber surface enables the bonding of PLA by functional group reaction, therefore improving the interfacial compatibility between fibers and PLA matrix. In this study, PLA and sisal fibers were melt-blended to fabricate bio-based composites via in situ reactive interfacial compatibilization with addition of an epoxy-functionalized oligomer. Figure 4 presents the illustration of interfacial compatibilization between PLA and SF via in situ reaction with the ADR oligomer during the melt-blending processing. ADR is a kind of multi-epoxy-functionalized oligomer [19,20,21], which can react with end group of PLA and hydroxyl groups on natural fiber surfaces. It might bond PLA molecules onto the fiber surface and play a hinge-like role between sisal fibers and the PLA matrix. Therefore, the addition of ADR can improve the interfacial compatibility of composites. The improved interfacial adhesion of PLA/SF/ADR composites can be observed in the above-mentioned SEM images.

### 3.2. FTIR Characterization

**Figure 5:**
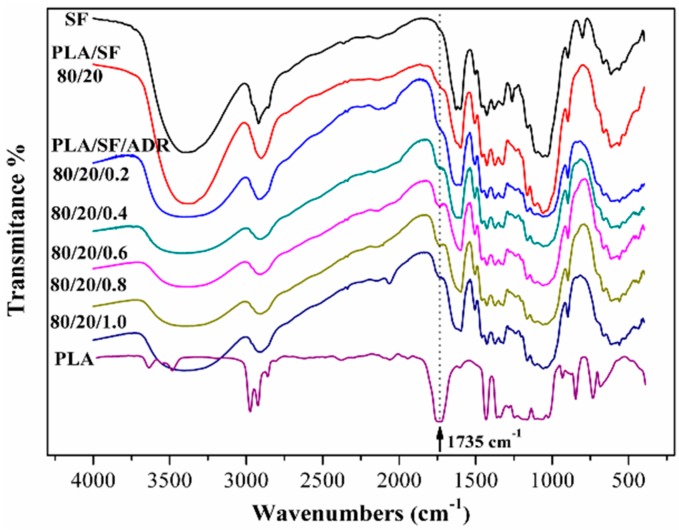
The FTIR spectra of alkaline-treated SF, extracted SF from the composites, and PLA resin.

To demonstrate the reaction of the ADR oligomer with PLA and SF, the FTIR spectra of alkaline-treated SF and extracted SF from corresponding composites are shown in Figure 5. The peak at 1735 cm^−1^ represents the stretching vibration peak of carbonyl (C=O) groups. The absence of this 1735 cm^−1^ peak in alkaline-treated SF showed the alkaline treatment removed the lignin, impurities, pectin, and waxy substances of sisal fibers [16,17]. It was also observed that no obvious 1735 cm^−1^ peak occurred in the SF extracted from PLA/SF composites, reflecting the poor interfacial interaction between SF and PLA matrix in PLA/SF composites. However, it was found that the 1735 cm^−1^ peak was enhanced with the addition of ADR into PLA/SF composites, especially for PLA/SF/ADR composites with 0.4, 0.6, and 0.8 wt % ADR addition. This phenomenon indicated the reaction of the ADR oligomer with PLA and SF during the melt-blending processing, which resulted in the bonding of PLA molecular chains to sisal fibers.

### 3.3. DSC Thermal Behaviors

**Figure 6:**
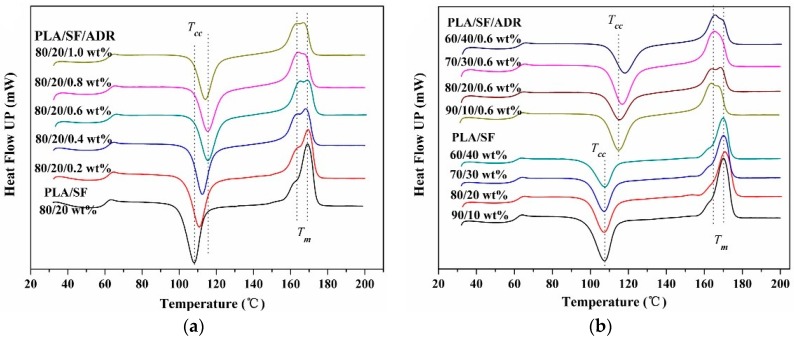
Differential scanning calorimetry (DSC) thermograms of PLA/SF composites and PLA/SF/ADR composites with different ADR addition (**a**) and different SF content (**b**) at the second heating scan with a rate of 10 °C/min.

Figure 6a shows the DSC thermograms of PLA/SF composites and PLA/SF/ADR composites with constant 20 wt % SF and different ADR addition, from 0.2 wt % to 1.0 wt %, at the second heating scan. A cold crystallization peak and melting peak occurred in Figure 6a. At the second heating scan, as the temperature elevated, the frozen PLA chain segments regain movement ability, resulting in the presence of cold crystallization. It can be seen from Figure 6a that the cold crystallization temperature (*T_cc_*) of PLA/SF/ADR composites shifts to a higher temperature region compared with PLA/SF composites. The cold crystallization temperatures (*T_cc_*) for PLA/SF and PLA/SF/ADR composites with different ADR additions are listed in Table 2. The *T_cc_* was improved with the addition of ADR oligomer, which meant that PLA/SF/ADR composites need higher temperature to regain chain segments movement ability in comparison with that of PLA/SF composites. Therefore, this phenomenon demonstrated the restricted chain movement ability of PLA/SF/ADR composites [27]. This result can be ascribed to the improved interfacial interaction between PLA and SF via addition of ADR. However, the *T_cc_* of PLA/SF/ADR (80/20/1.0) composites was 114.1 °C, which was reduced compared with that of PLA/SF/ADR (80/20/0.8) composites (115.5 °C), which implied that excessive addition of ADR might be detrimental to the connecting effect of ADR between SF and PLA matrix.

At the same time, it was also found that with the addition of ADR oligomer, an obvious shoulder peak appeared in the left of the melting endothermal peak (*T_m_* 169.5 °C) for PLA/SF/ADR composites, which indicated the formation of poor crystalline regions within PLA [28,29], corresponding to lower melting temperature. The improved interfacial interaction of PLA/SF/ADR composites resulted in restricted molecular chain movement of PLA and then more poor crystalline regions formed in the crystal growth stage, therefore a shoulder peak corresponding to lower melting temperature occurred for PLA/SF/ADR composites. The presence of a peak shoulder might also be ascribed to the formation of different crystalline structures within PLA, referred to as transcrystallinity [15].

To further confirm these results, Figure 6b presents the DSC thermograms of PLA/SF composites and PLA/SF/ADR composites with different SF content at the second heating scan. The cold crystallization temperatures are presented in Table 3. For PLA/SF/ADR composites, the *T_cc_* increased considerably in comparison with PLA/SF composites, and an obvious shoulder peak arose in the left of the melting endothermal peak. These results were consistent with Figure 6a. At the same time, it was found that the change of SF content had little effect on *T_cc_* of PLA/SF composites. However, the *T_cc_* of PLA/SF/ADR composites improved with increasing SF content. For PLA/SF composites, poor interfacial interaction between SF and PLA matrix revealed that excessive addition of SF has little effect on the movement of PLA chain segments. By contrast, for PLA/SF/ADR composites, the more SF was added, the more the sisal fibers participated in the interfacial reaction. As a result, the improved interfacial interaction between SF and the PLA matrix caused more severe restriction of PLA chain segment movement and the *T_cc_* of PLA/SF/ADR composites increased. 

**Figure 7:**
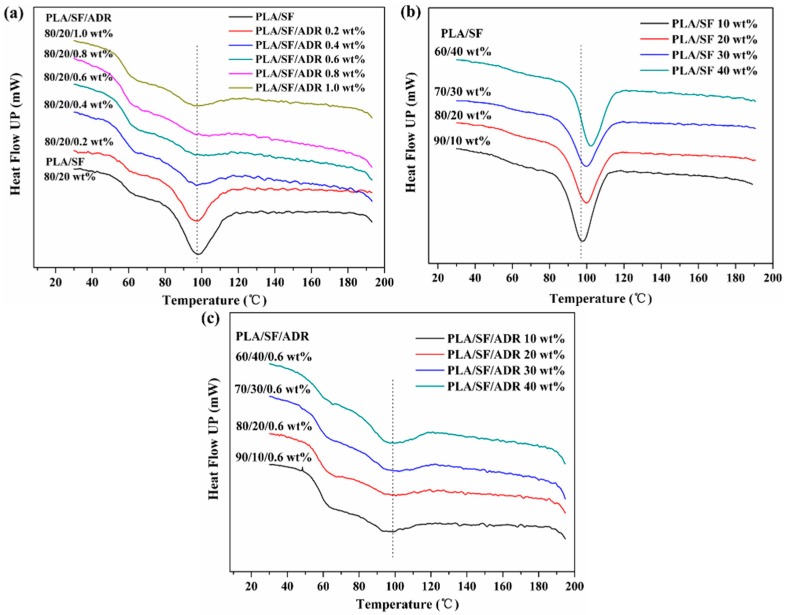
DSC thermograms of PLA/SF composites and PLA/SF/ADR composites with different ADR addition (**a**) and different SF content (**b**,**c**) at a cooling rate of 5 °C/min.

The DSC thermograms of PLA/SF composites and PLA/SF/ADR composites with different ADR addition and different SF content at a cooling rate of 5 °C/min are shown in Figure 7. It was found that the cooling crystallization exothermal peak declined with the addition of ADR oligomer (Figure 7a). In terms of previous analyses, the incorporation of ADR oligomer into PLA/SF composites improved the interfacial interaction between SF and PLA matrix, thus the movement of PLA segments were restricted, especially in the PLA matrix close to the interface. In the crystal growth stage during cooling, the mobility of polymer segments is significant for crystallization. It can be deduced that during the cooling process, no more PLA chain segments participated in cooling crystallization with the further addition of oligomer ADR due to restricted movement of the PLA chain segments, therefore the crystallization of PLA/SF/ADR composites declined. Similar crystallization behaviors were found in PLA/SF composites and PLA/SF/ADR composites with different SF content (Figure 7b,c). The cooling crystallization exothermal peaks of PLA/SF/ADR composites were significantly decreased compared with that of PLA/SF composites. At the same time, it was found that the crystallization temperature improved as SF content increased, corresponding to an enhanced crystal nucleation ability with greater addition of SF. This can be attributed to the heterogeneous nucleation effect of SF.

**Figure 8:**
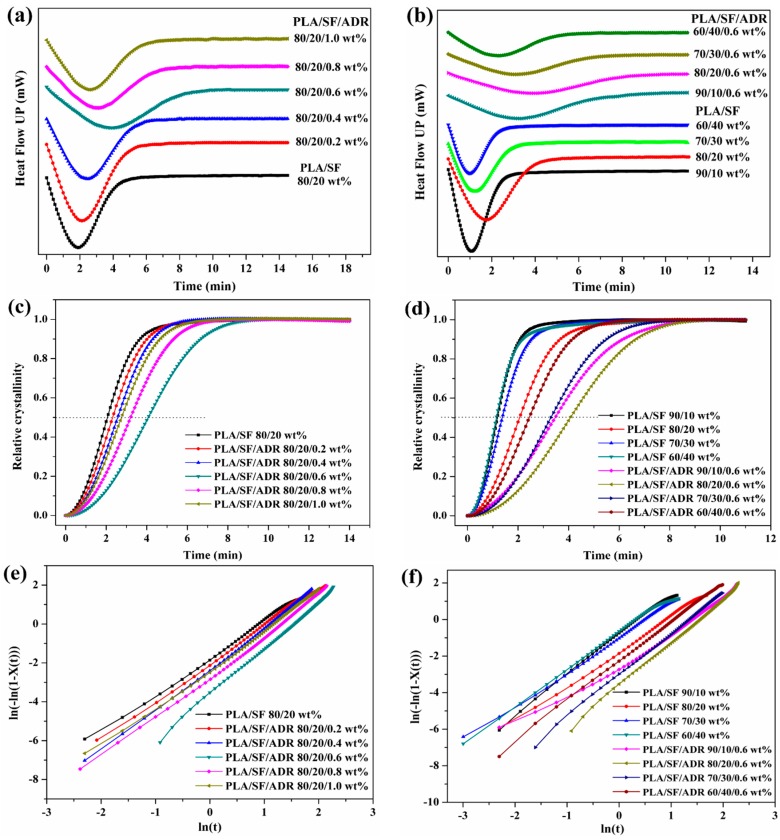
Isothermal crystallization of PLA/SF and PLA/SF/ADR composites: DSC thermograms (**a**,**b**); relative crystallinity (**c**,**d**) and Avrami plots (**e**,**f**).

The isothermal crystallization behavior of PLA/SF composites and PLA/SF/ADR composites was investigated at 110 °C. Figure 8a,b illustrate the isothermal crystallization thermograms of PLA/SF composites and PLA/SF/ADR composites with different ADR addition and different SF content at 110 °C. The thermograms recorded heat flow (*dH*/*dt*) as a function of time. From the isothermal crystallization thermograms, the relative crystallinity as a function of crystallization time can be calculated by the following equation:

(1) $$X_{t}=\frac{Q_{t}}{Q_{\infty }}=\frac{\int_{0}^{t}\left(dH/dt\right)dt}{\int_{0}^{\infty }\left(dH/dt\right)dt}$$

where *X*(*t*) is the relative crystallinity, and *Q_∞_* and *Q_t_* are the heat generated at infinite time and at time *t*. Figure 8c,d presents the change in relative crystallinity as a function of crystallization time. To further analyze the crystallization kinetics of composites, a classic Avrami equation was used [30,31].

(2) $$X\left(t\right)=1-exp\left(-kt^{n}\right)$$

where *X(t)* is the relative crystallinity, *k* is the crystallization rate constant, and *n* is the Avrami exponent reflecting the mechanisms of crystal nucleation and growth, and the following equation can be deduced from Equation (2)

(3) ln{−ln[1−X(t)]}=lnk+nlnt

By plotting ln [−ln(1 − *X(t)*)] versus ln(*t*), the Avrami exponent, *n*, and crystallization rate constant, *k*, were determined. Meanwhile, the crystallization half-time *t*_1/2_, reflecting the crystallization rate, can be calculated with the below equation:

(4) $$t_{1/2}={\left(\frac{\ln2}{k}\right)}^{1/n}$$

The obtained Avrami parameters of *n* and *k*, and calculated *t*_1/2_ are summarized in Table 4 and Table 5.

For PLA/SF and PLA/SF/ADR composites with different ADR addition (Figure 8a), the isothermal crystallization rate decreased with the addition of ADR oligomer. As can be seen from Table 4, the crystallization half-time is 2.04 min for PLA/SF composites, and *t*_1/2_ increased with addition of ADR. The crystallization half-time increased to 3.99 min for composites with 0.6 wt % ADR. However, *t*_1/2_ tended to decrease for composites with 0.8 wt % and 1.0 wt % ADR, for which the *t*_1/2_ values were 3.09 min and 2.68 min, respectively. The calculated crystallization half-time was consistent with the crystallization thermograms. In terms of the analyses mentioned above, the incorporation of ADR into PLA/SF composites improved the interfacial interaction between SF and PLA, but it also restricted the movement of the PLA chain segments. The decline in the crystallization rate of the composites with addition of ADR can be credited to the restricted movement of the PLA chain segments. The ADR oligomer connected PLA to the fiber surface via in situ reaction. However, it was found that excessive addition of ADR was detrimental to the bonding effect of ADR between SF and the PLA matrix. The increased crystallization rate of composites with 0.8 and 1.0 wt % ADR addition can be ascribed to the decline of the bonding effect of the ADR oligomer. In addition, ADR is a kind of oligomer with low molecular weight, and it might plasticize the PLA matrix in excessive quantities, thus facilitating chain segment movement and improving the crystallization rate. It was found that for PLA/SF composites and PLA/SF/ADR composites with different ADR addition, the *n* value varied from 1.954 to 2.341, reflecting that the crystals tended to grow in two dimensions.

For PLA/SF composites and PLA/SF/ADR composites with different SF content (Figure 8b), a similar crystallization behavior occurred. The addition of 0.6 wt % ADR decreased the crystallization rate for each SF content, from 10 wt % to 40 wt %. It was also found that as the SF content increased, the crystallization half-time improved, then decreased significantly (see Table 5). This phenomenon occurred in both PLA/SF composites and PLA/SF/ADR composites with different SF content. These results are related to the heterogeneous nucleation effect of SF. Excessive addition of SF restricted the movement of PLA chain segments; meanwhile, it provided more heterogeneous nucleation points. The enhanced heterogeneous nucleation resulted in increase in the crystallization rate of composites with high SF content (30 wt % and 40 wt %).

### 3.4. Dynamic Rheological Behaviors

The viscoelastic response of polymers and polymer composites are very sensitive to changes of microstructure, including chain structure and phase morphology, which has attracted fundamental interest in this study. Dynamic small amplitude oscillatory shear (SAOS) was performed to probe the effect of melting reactive processing with ADR oligomer on the microstructure of PLA/SF composites.

**Figure 9:**
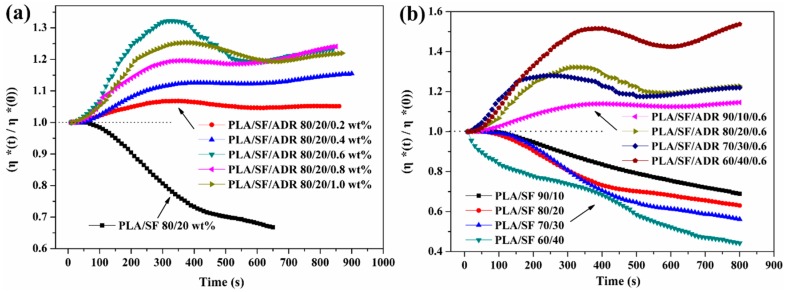
Relative complex viscosity (η*(t)/η*(0)) versus time of PLA/SF and PLA/SF/ADR composites with different ADR addition (**a**) and different SF content (**b**).

To detect the in situ reaction of composite melts, dynamic time sweep tests were performed in this study. The relative complex viscosity (η*(t)/η*(0)) versus time of PLA/SF composites and PLA/SF/ADR composites with different ADR addition are shown in Figure 9a. It was found that the complex viscosity of PLA/SF (80/20 wt %) composites decreased dramatically within the time sweep because of thermal degradation. However, the presence of ADR oligomers counteracted the degradation of the composite melts. The relative complex viscosity even increased at the early stage of the time sweep. This phenomenon can be ascribed to the improved interfacial interaction between SF and the PLA matrix via in situ reaction of ADR oligomers with SF and PLA, and meanwhile, the chain extension effect of ADR on PLA also contributed to the improvement of complex viscosity. Furthermore, it was found that the η*(t)/η*(0) value of PLA/ADR/ADR (80/20/0.6 wt %) composites possess the most significant improvement in the early stage of the time sweep. These results were consistent with the analyses mentioned above. Excessive addition of ADR oligomer, more than 0.6 wt % content, did not improve the interfacial interaction of the composites.

**Figure 10:**
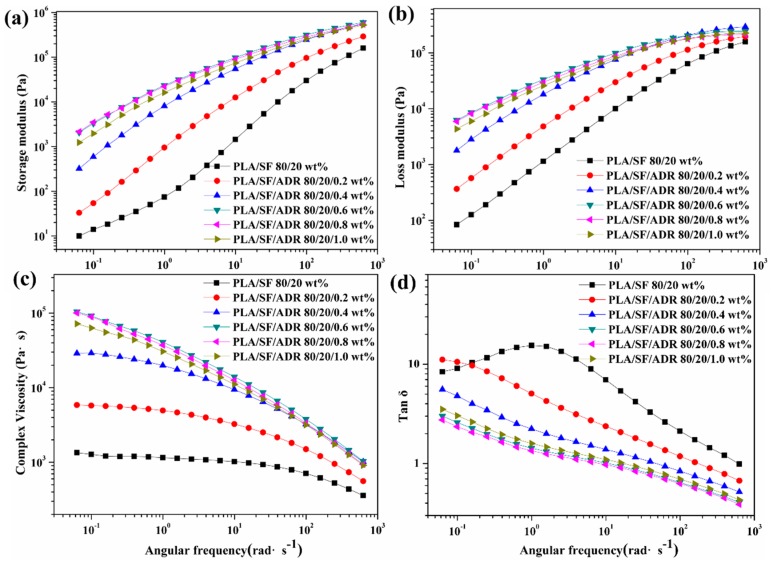
Changes in storage modulus (**a**); loss modulus (**b**); complex viscosity |η*| (**c**), and tan *δ* (**d**) as functions of angular frequency for PLA/SF and PLA/SF/ADR composites with different ADR addition.

Figure 9b shows the relative complex viscosity (η*(t)/η*(0)) versus time of PLA/SF composites and PLA/SF/ADR composites with different SF content. For PLA/SF composites with different SF content, the complex viscosity declined severely within the time sweep. The addition of 0.6 wt % ADR oligomer effectively inhibited the reduction of the complex viscosity of the composites. As can be seen from Figure 10b, the η*(t)/η*(0) value of PLA/SF/ADR composites tended to be enhanced within the time sweep. Furthermore, it was found that for PLA/SF composites, the more SF was added, the more the η*(t)/η*(0) value would decrease. That is because the presence of SF aggravated the thermal degradation of composites. However, for PLA/SF/ADR composites with 0.6 wt % ADR oligomer, the η*(t)/η*(0) value of composites increased with increasing SF content because more SF addition could improve the interfacial interaction between SF and the PLA matrix. The PLA/SF/ADR (60/40/0.6 wt %) composites exhibited the greatest improvement in the η*(t)/η*(0) value.

The changes of storage modulus (G′), loss modulus (G″), complex viscosity |η*| and loss tangent as functions of angular frequency for PLA/SF and PLA/SF/ADR composites with different ADR addition are shown in Figure 10. It can be seen from Figure 10a,b that the addition of ADR oligomer improved the storage modulus and loss modulus of PLA/SF composites. It reflects the enhanced viscoelastic response of PLA/SF/ADR composites compared with that of PLA/SF composites. Figure 10c presents the changes of complex viscosity as a function of angular frequency. It can be seen that the complex viscosity improved with addition of ADR oligomer, especially in the low angular frequency region. In addition, the pattern of dependence of complex viscosity on frequency (the change tendency of complex viscosity with frequency) varied to some extent due to the addition of ADR. For PLA/SF composites and PLA/SF/ADR composites with low ADR content (0.2 wt % and 0.4 wt %) the complex viscosity remained nearly constant in the low angular frequency region, and then decreased as angular frequency increased. This transition from the Newtonian plateau to the power law regime at the inflection point is a typical phenomenon of polymer materials. However, it was found that the Newtonian plateau region decreased with the addition of ADR oligomer. For composites with high ADR content (0.6 wt %, 0.8 wt %, and 1.0 wt %), the shear-thinning behavior occurred in the whole angular frequency region, which more obviously exhibited the power law regime [32,33]. The enhanced shear-thinning behavior of PLA/SF/ADR composites can be ascribed to the improved interfacial interaction of composites. For PLA/SF/ADR composites, for which the interfacial interaction between SF and PLA matrix were enhanced, the sisal fibers can act as physical crossing points in composite melts. Therefore, it can be deduced that more significant disentanglement of the polymer chain occurred as the angular frequency increased, resulting in more obvious shear-thinning behavior than that of PLA/SF composites. Figure 10d presents the changes of loss tangent (tan *δ* = G″/G′) as a function of angular frequency. It can be seen that the tan *δ* of PLA/SF/ADR composites decreased in comparison with that of PLA/SF composites. The PLA/SF/ADR composites possess a more dramatically elastic response. The frequency dependence of the loss tangent can be used to characterize the gel point of the cross-linking system [34,35]. This method can be adopted for polymer composites to evaluate the percolation thresholds of the fillers [36,37]. The tan *δ* of PLA/SF/ADR composites displayed less dependence on the angular frequency, which exhibited a gel-like behavior. In terms of the analyses mentioned above, the presence of ADR improved the interfacial interaction of composites, and the physical crossing points of sisal fibers in composite melts. Therefore, the stress relaxation of the PLA molecular chain became more difficult in the melt state; the elastic response of the PLA/SF/ADR composite melt was enhanced and tended to exhibit a gel-like behavior.

### 3.5. DMA Characterization

**Figure 11:**
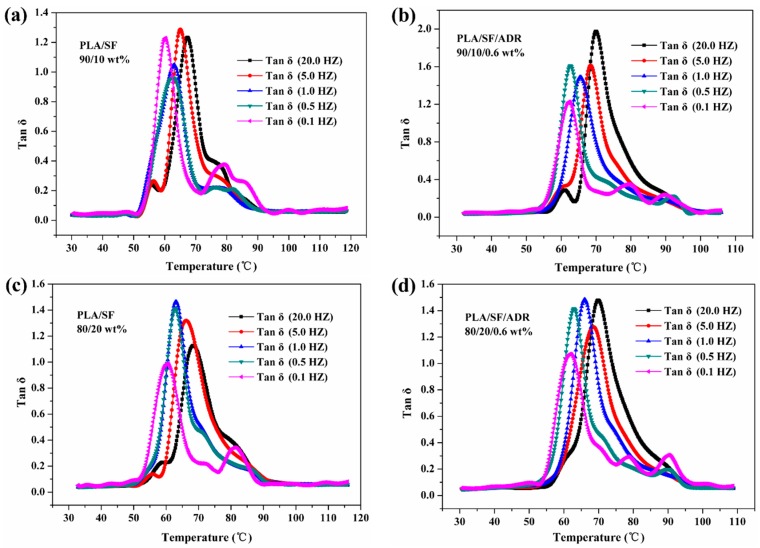

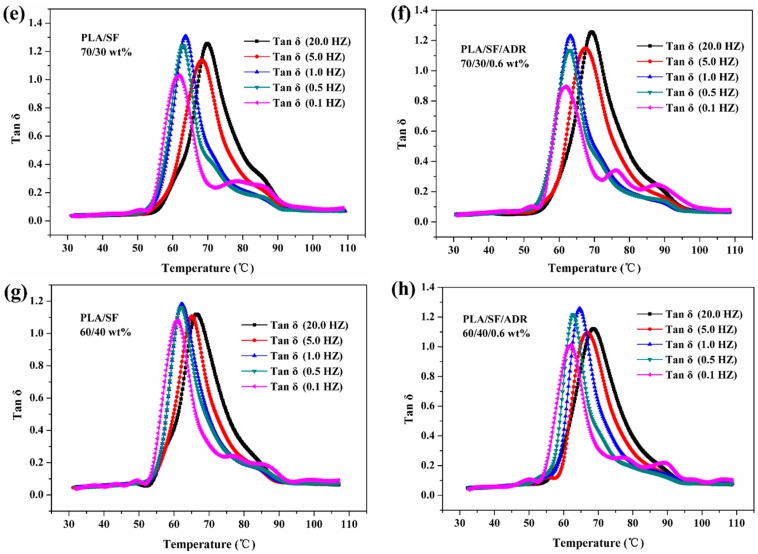
Effect of different frequencies on tan *δ* of PLA/SF and PLA/SF/ADR composites with different SF content at a heating rate of 2 °C/min.

In order to evaluate the dynamic mechanical properties and investigate the effect of ADR on the glass transition of composites, the DMA measurements of PLA/SF composites and PLA/SF/ADR composites with different SF content were carried out at different frequencies. The effect of different frequencies on the loss tangent (tan *δ*) of PLA/SF and PLA/SF/ADR composites with different SF content with a heating rate of 2 °C/min is shown in Figure 11. It was observed that in the whole temperature range, the α relaxation for PLA/SF and PLA/SF/ADR composites was between 60 °C and 70 °C which corresponds to the glass transition temperature (*T_g_*) of the composites. The glass transition temperature of the composites is summarized in Table 6. The *T_g_* of the composites increased as the frequencies elevated. It was found that the *T_g_* of PLA/SF/ADR composites was improved compared with that of PLA/SF composites at the same test frequencies, which demonstrated that PLA chain segment mobility was restrained. This result confirmed the enhanced interfacial interaction between SF and the PLA matrix via in situ interfacial reaction.

To further analyze the glass transition of the composites, the activation energies of glass transition relaxation (∆*E*_a_) were calculated based on the dependence of *T_g_* on frequency. The ∆*E*_a_ represents the energy barrier of glass transition relaxation. It can be adopted to characterize the relationship between polymer chain segment mobility and the time scale. Five different test frequencies were used in this study (0.1, 0.5, 1, 5, and 20 HZ). The changes of glass transition temperature were interrelated with test frequencies using the Arrhenius equation [38,39]. On the basis of classic Arrhenius equation, the molecular relaxation time can be expressed as:

(5) $$\tau =\tau_{0}e^{\frac{\Delta E-\gamma \sigma }{RT}}$$

where ∆*E* and *σ* are the activation energy of relaxation and the stress, respectively, *τ*_0_ is the hypothetical relaxation time at an infinite temperature, *T* is the absolute temperature, *γ* is the variable and R is the gas constant.

Here, the stress (*σ*) is small, thus Equation (5) can be simplified as:

(6) $$\tau =\tau_{0}e^{\frac{\Delta E}{RT}}$$

(7) $$\ln\tau =\ln\tau_{0}+\frac{\Delta E}{RT}$$

The relaxation times were calculated from the relationship:

(8) $$\tau =\frac{1}{f}$$

A combination of Equations (7) and (8) leads to:

(9) $$\ln f=-\ln\tau_{0}-\frac{\Delta E}{RT}$$

**Figure 12:**
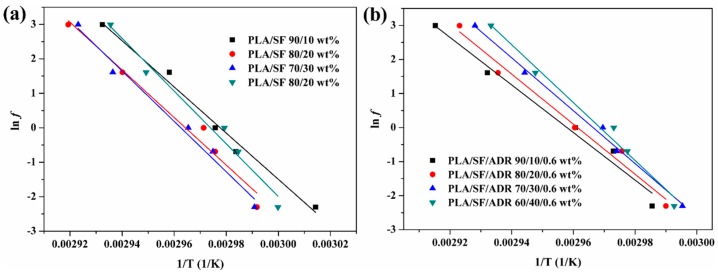

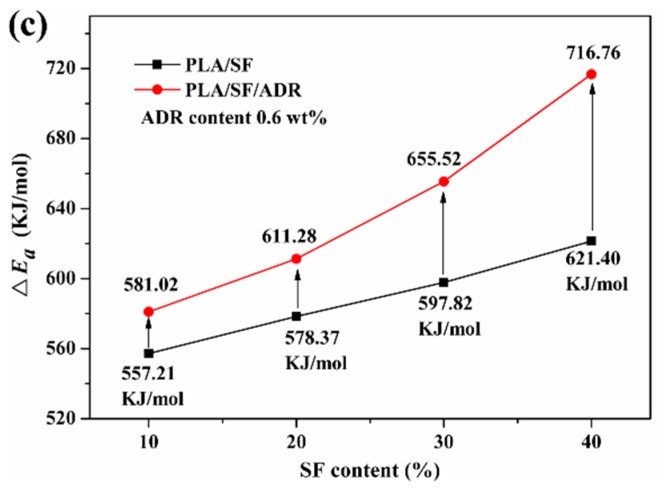
Arrhenius plots of relaxation times versus 1/T and the respective linear fits of PLA/SF composites (**a**) and PLA/SF/ADR composites (**b**) with different SF content and the calculated activation energies (∆*E*_a_) of glass transition relaxation (**c**).

According to Equation (9), a plot of ln*f* versus 1/*T* should give a straight line with a slope that is proportional to activation energy associated to the glass transition relaxation. Figure 12 shows the Arrhenius plots of relaxation times versus 1/*T*, the respective linear fits of PLA/SF composites and PLA/SF/ADR composites with different SF content, and the calculated activation energies of glass transition relaxation. As can be seen from Table 7 and Figure 12c, the calculated ∆*E*_a_ of composites increased with the addition of ADR oligomer at equal SF content, which implied that the improved interfacial adhesion of composites increased the energy barrier of the mobility of the PLA chain segments. In addition, for both PLA/SF composites and PLA/SF/ADR composites, the ∆*E*_a_ value increased with increasing SF content. Furthermore, the improvement in activation energy of PLA/SF/ADR composites compared with that of PLA/SF composites at the identical SF content was elevated with increased SF content (see Figure 12c). The greater the addition of SF, the more sisal fibers participated in interfacial reaction, resulting in increased restriction of PLA chain segments. These results are in accordance with the thermal and rheological analyses mentioned above.

### 3.6. Mechanical Properties

**Figure 13:**
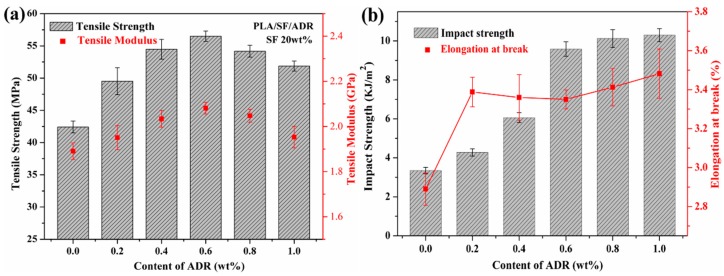
Tensile strength and tensile modulus (**a**), elongation at break and impact strength (**b**) of PLA/SF/ADR composites with different ADR addition and constant 20 wt % SF content.

**Figure 14:**
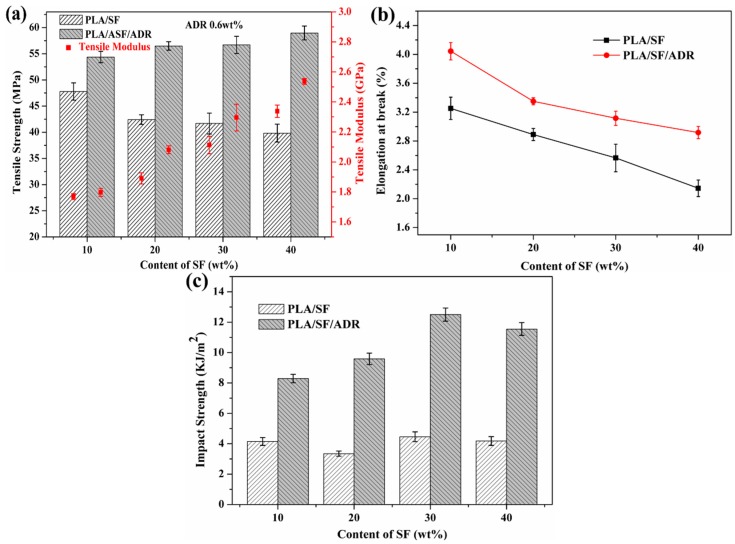
Tensile strength and tensile modulus (**a**); elongation at break (**b**), and impact strength (**c**) of PLA/SF and PLA/SF/ADR composites with different SF content and constant 0.6 wt % ADR addition.

Figure 13 shows the tensile properties and impact strength of PLA/SF composites and PLA/SF/ADR composites with different ADR addition. It was found that the addition of ADR oligomer improved the tensile strength of the composites (Figure 13a). Moreover, as the ADR content increased, the tensile strength of PLA/SF/ADR composites increased at first and then decreased. The PLA/SF/ADR (80/20/0.6 wt %) composites possessed the highest tensile strength. A similar variation tendency occurred for the tensile modulus of the composites, which reflected the enhanced stiffness of PLA/ADR/SF composites compared with that of PLA/SF composites. Furthermore, it was found that the elongation at break and the impact strength of PLA/SF/ADR composite were improved in comparison with that of PLA/SF composites (Figure 13b), which indicated an enhanced toughness of composites with addition of ADR oligomer. These results demonstrated that the incorporation of ADR oligomers simultaneously reinforce and toughen the PLA/SF composites. Figure 14 shows the tensile properties and impact strength of PLA/SF composites and PLA/SF/ADR composites with different SF content. For PLA/SF composites, the tensile strength decreased as SF content increased. However, for PLA/SF/ADR composites with 0.6 wt % ADR oligomer, the tensile strength improved with the increase of SF content, exhibiting that the greater the addition of SF, the greater the tensile strength compared with that of PLA/SF composites at the identical SF content (Figure 14a). In addition, the tensile modulus of PLA/SF and PLA/SF/ADR composites were enhanced with high SF content, and the elevation of tensile modulus became greater at higher SF content. Figure 14b presents that the elongation at break of the composites decreases with increasing SF content, and at the identical SF content the elongation at break is improved via addition of ADR oligomer. The impact strength of PLA/SF/ADR composites with different SF content was also improved in comparison with that of PLA/SF composites (see Figure 14c).

In brief, when the fiber-reinforced plastic is bearing force, the role of the interface is to transmit stress from the polymer matrix to the fibers; therefore, the mechanical properties of PLA/SF composites in this study were significantly influenced by fiber–matrix interaction [40]. According to the microstructure characterization and analyses mentioned above, the addition of ADR oligomer improved the interfacial adhesion of PLA/SF composites via in situ interfacial reaction during melt-blending and processing. The improved interfacial interaction of PLA/SF composites enhanced the efficiency of transmitting stress from the PLA matrix to sisal fibers, thus improving the mechanical properties. However, excessive addition of ADR was detrimental to the connecting effect of ADR between SF and the PLA matrix, which did not facilitate the enhancement of interfacial adhesion. Therefore, the interfacial adhesion of PLA/SF/ADR composites with high ADR addition (0.8 wt % or 1.0 wt %) slightly decreased as compared with composites with 0.6 wt % ADR. ADR is a kind of oligomer with lower molecular weight and a more flexible chain compared with PLA. It had a plasticizing effect on a PLA matrix, which also contributed to the toughening of composites.

At the same time, poorer interfacial adhesion resulted in more microstructural defects of the composites (Figure 1, Figure 2 and Figure 3). The microstructure defects would cause stress concentration points when the composites bearing force, which were bad for the mechanical properties of composites. In addition, the low interfacial bonding of PLA/SF composites permitted composites to debond facilely. These factors resulted in forward breakage of PLA/SF composite samples compared with that of PLA/SF/ADR composites in tensile tests. The elongation at break of PLA/SF/ADR composites were improved compared with that of PLA/SF composites. 

Improving the interfacial interaction between SF and the PLA matrix contributed to the formation of a moderate interface in the composites. A moderate interface can effectively relieve the stress concentration under loading conditions, and transmit stress uniformly from the matrix to the fibers [41,42]. For PLA/SF composites with different SF content, the greater the addition of sisal fibers, the more microstructural defects were present in the composites. This resulted in a decline in the mechanical properties of PLA/SF composites with increased SF content. By contrast, the mechanical properties of PLA/SF/ADR composites were improved because of enhanced interfacial adhesion of the composites via in situ reactive interfacial compatibilization by the addition of ADR oligomers.

## 4. Conclusions

In this study, polylactide/sisal fibers biocomposites were fabricated using a simple in situ reactive melt-blending method with the addition of an epoxy-functionalized oligomer. ADR oligomer played a hinge-like role between sisal fibers and PLA matrix during melt blending and processing, resulting in improved interfacial interaction between SF and the PLA matrix. SEM morphology characterization confirmed the improved interfacial adhesion of PLA/SF/ADR composites, and FTIR analysis demonstrated the bonding of the PLA molecular chain with SF. The effects of in-situ reaction with ADR oligomer on the thermal and rheological properties of PLA/SF composites were investigated. The results indicated that the crystallization ability of composites declined and the viscoelastic response was enhanced with the addition of ADR. In turn, these results further reflected the restricted mobility of the PLA molecular chains because of the enhanced interfacial interaction with SF. The activation energies of glass transition relaxation (∆*E*_a_) of the composites calculated by dynamic mechanical analysis justified the results as well. The tensile properties and impact strength of composites were improved because of the improved interfacial compatibility, which demonstrated that the incorporation of ADR oligomers could simultaneously reinforce and toughen the PLA/SF composites.

## Figures and Tables

**Table 1:** Properties of sisal fibers.

Fiber Diameter (μm)	Fiber Density (g/cm^3^)	Cellulose Content (%)	Hemicellulose Content (%)	Lignin Content (%)
25–200	1.45	67–78	10–14	8–11

**Table 2:** The cold crystallization temperatures (*T_cc_*) for PLA/SF and PLA/SF/ADR composites with different ADR additions.

Composites	PLA/SF	PLA/SF/	PLA/SF/	PLA/SF/	PLA/SF/	PLA/SF/
		ADR	ADR	ADR	ADR	ADR
	80/20	80/20/0.2	80/20/0.4	80/20/0.6	80/20/0.8	80/20/1.0
*T_cc_* (°C)	107.7	110.8	112.1	115.7	115.5	114.1

**Table 3:** The cold crystallization temperatures (*T_cc_*) for PLA/SF and PLA/SF/ADR composites with different SF content.

Composites	*T_cc_* (°C)	Composites	*T_cc_* (°C)
PLA/SF 90/10 wt %	107.1	PLA/SF/ADR 90/10/0.6 wt %	115.4
PLA/SF 80/20 wt %	107.5	PLA/SF/ADR 80/20/0.6 wt %	115.7
PLA/SF 70/30 wt %	107.4	PLA/SF/ADR 70/30/0.6 wt %	116.9
PLA/SF 60/40 wt %	107.4	PLA/SF/ADR 60/40/0.6 wt %	117.8

**Table 4:** Isothermal crystallization half time and kinetic parameters of PLA/SF/ADR composites with different ADR addition.

Samples	*n*	*k* (min^−n^)	*t*_1/2_ (min)
PLA/SF 80/20 wt %	1.954	0.172	2.04
PLA/SF/ADR 80/20/0.2 wt %	2.030	0.139	2.21
PLA/SF/ADR 80/20/0.4 wt %	2.159	0.0965	2.49
PLA/SF/ADR 80/20/0.6 wt %	2.341	0.0272	3.99
PLA/SF/ADR 80/20/0.8 wt %	2.159	0.0604	3.09
PLA/SF/ADR 80/20/1.0 wt %	2.094	0.0879	2.68

**Table 5:** Isothermal crystallization half time and kinetic parameters of PLA/SF and PLA/SF/ADR composites with different SF content.

Samples	*n*	*k* (min^−*n*^)	*t*_1/2_ (min)
PLA/SF 90/10 wt %	2.218	0.464	1.20
PLA/SF 80/20 wt %	1.954	0.172	2.04
PLA/SF 70/30 wt %	1.933	0.373	1.38
PLA/SF 60/40 wt %	2.041	0.506	1.17
PLA/SF/ADR 90/10/0.6 wt %	1.921	0.0678	3.35
PLA/SF/ADR 80/20/0.6 wt %	2.341	0.0272	3.99
PLA/SF/ADR 70/30/0.6 wt %	2.245	0.0491	3.25
PLA/SF/ADR 60/40/0.6 wt %	2.141	0.105	2.41

**Table 6:** The glass transition temperature of PLA/SF composites and PLA/SF/TGIC composites with different ADR addition at different frequencies of tan *δ*.

Samples	Temperature *T_g_* (°C)				
	0.1 (HZ)	0.5 (HZ)	1 (HZ)	5 (HZ)	20 (HZ)
PLA/SF 90/10 wt %	58.6	62.0	62.9	64.9	67.9
PLA/SF/ADR 90/10/0.6 wt %	61.8	63.2	64.6	67.9	69.9
PLA/SF 80/20 wt %	61.1	62.9	63.4	67.0	69.4
PLA/SF/ADR 80/20/0.6 wt %	61.3	62.9	64.6	67.5	68.95
PLA/SF 70/30 wt %	61.2	63.0	63.1	66.4	68.9
PLA/SF/ADR 70/30/0.6 wt %	60.7	63.1	63.6	66.5	68.4
PLA/SF 60/40 wt %	60.2	61.9	62.5	65.9	67.5
PLA/SF/ADR 60/40/0.6 wt %	61.0	62.7	63.2	66.1	67.8

**Table 7:** The calculated activation energies of glass transition relaxation for PLA/SF composites and PLA/SF/ADR composites with different SF content.

Samples	PLA/SF	PLA/SF	PLA/SF	PLA/SF	PLA/SF/	PLA/SF/	PLA/SF/	PLA/SF/
	90/10	80/20	70/30	60/40	ADR	ADR	ADR	ADR
					90/10/0.6	80/20/0.6	70/30/0.6	60/40/0.6
∆*E*_a_ (KJ/mol)	557.21	578.37	597.82	621.40	581.02	611.28	655.52	716.76
